# Establishment of semen collection technique using electroejaculator and semen cryopreservation of Javan leopard (*Panthera pardus melas* Cuvier, 1809)

**DOI:** 10.14202/vetworld.2021.3156-3163

**Published:** 2021-12-25

**Authors:** Bongot Huaso Mulia, Ardyta Widianti, Jansen Manansang, Dedi Rahmat Setiadi, Vincentia Trisna Yoelinda, Taufiq Purna Nugraha, Ni Wayan Kurniani Karja, Raden Iis Arifiantini

**Affiliations:** 1 Biology Reproductive Program, Post Graduate School, IPB University, Jl. Raya Dramaga, Dramaga Campus, Bogor 16680, Indonesia; 2 Department of Life Sciences, Taman Safari Indonesia, Jalan Kapten Harun Kabir No.724 Cisarua 16750, Bogor, West Java, Indonesia; 3 Department of Clinic, Reproduction, and Pathology, Faculty of Veterinary Medicine, IPB University, Jl. Agatis, Dramaga Campus, Bogor 16680, Indonesia; 4 Zoological Division, Research Center for Biology, Indonesian Institute of Science, Cibinong 16911 West Java, Indonesia

**Keywords:** electroejaculator, Javan leopard, semen characteristics, semen cryopreservation

## Abstract

**Background and Aim:**

The Javan leopard (*Panthera pardus melas* Cuvier, 1809) is a subspecies of *Panthera pardus* spp., spread across the African and Asian regions. Information on reproductive aspects is crucial for wild animals, including the Javan leopard. In this study, we aimed to developelectroejaculator (EE) techniques and evaluate cryopreservation success in Javan leopard semen.

**Materials and Methods:**

The semen of four adult Javan leopards was collected once a week using EE. Placement of the EE probe in the rectum was performed after ultrasound imaging (ultrasonography) to determine the prostate body location. The semen obtained was then evaluated macroscopically and microscopically. Three Javan leopards were used for cryopreservation. The ejaculate was divided into two parts [i.e., one part diluted with AndroMed^®^ (Minitüb, Tiefenbach, Germany) and the other part with Steridyl^®^(Minitüb, Tiefenbach, Germany)] at a 1:1 ratio immediately after collection and evaluation. The semen was then packed in a 0.25 mL MiniStraw^®^ (Minitüb, Tiefenbach, Germany) then equilibrated at 4°C for 2 h. After equilibration, the straw was then frozen in liquid nitrogen vapor. Frozen semen was then stored in containers until further evaluation.

**Results:**

The results showed that ejaculation response occurred at all levels of stimulation, while erections did not always occur. The fastest ejaculation and erection occurred at the fourth voltage. The macroscopic evaluation showed that the semen volume was 0.80±0.26 mL, cloudy white, pH 7.44±0.14, and with watery semen consistency. The microscopic evaluation showed that the sperm motility was 66.98±0.39%, with sperm viability of 75.6±1.79%. Sperm concentration was 62.17±46.95×10^6^ mL^−1^ with a total concentration of 42.14±23.51×10^6^ cells. Normal sperm morphology is only 40.72±6.26%.

**Conclusion:**

This study concluded that the development of a semen collection technique using an EE preceded by imaging of the EE probe location using ultrasound was effective for the ejaculation of Javan leopards. The characteristics of the semen of the Javan leopard showed moderate semen volume, sperm motility, and viability. Javan leopard showed low sperm concentration and normal sperm morphology.

## Introduction

The Javan leopard (*Panthera pardus melas* Cuvier, 1809) is a separate subspecies of nine *Panthera pardus* spp. The primary color of a leopard’s skin varies from golden yellow to black. Black spots in this animal are arranged in a rosette or rose-like pattern, and a tendency for melanism (black color) exists in this species [1]. A leopard with melanism is a black panther, born from the same mother as a leopard with a rosette pattern disguised in black fur. However, Javan leopard is currently experiencing extinction. The threat of Javan leopard extinction is caused by the decrease of habitat carrying capacity, the shrinking of natural forests on the island of Java, and the increasing conflict between the leopard and the community in the last decade [2]. Efforts to increase the population of the Javan leopard are regulated in the 2016-2026 action and conservation plan strategy, among others, by maximizing the management of the leopard population in *ex situ* as breeding stock. Increased participation of research institutions in producing data and information to increase knowledge and support Java leopard conservation efforts through research and scientific publications and public awareness has been noted [3].

Assisted reproductive technology is essential for wild cat conservation programs to increase population numbers and genetic variations [4]. The application of assisted reproductive technology requires sufficient knowledge of reproductive biology, including collection methods and semen characteristics. Studies on the reproductive biology of the Javan leopard have never been reported, unlike in several other wild cats, including the Indian [5], Arabian (*P. pardus nimr*) [6], and the Indochina (*P. pardus delacouri*) leopard [7]. Semen cryopreservation efforts in wild cats such as the jaguar (*Panthera onca*) [8], clouded leopard (*Neofelis nebulosa*) [9], *Prionailurus bengalensis euptilurus* [10], and the Sunda clouded leopard (*Neofelis diardi*) [11] have been reported. A sperm bank/genome bank is a vital step and effort for long-term wildlife conservation [12]. Moreover, semen cryopreservation of endangered animals is a crucial step to avoid species extinction [13]. Thus, assisted reproductive technology will assist in managing the genetic diversity of non-domestic cats separately managed at conservation institutions [14]. The semen to be cryopreserved must go through a semen extender process. A semen extender is an addition of materials to semen to maintain sperm viability [15].

Commercial extenders [e.g., AndroMed^®^ and Steridyl^®,^ (Minitüb, Tiefenbach, Germany)] are also available in markets aside from the homemade formula. Commercial extenders are well-known to be more practical both in field preparations and frozen straw productions purposes. In addition, commercial extenders can be used as semen extenders for domestic ruminants (e.g., cattle [16,17], goats [18], horses [19], and camels). Commercial extenders are also used for sperm cryopreservation of domestic cats [20]. Research on semen collection techniques, semen characteristics, and cryopreservation of the Javan leopard has never been done; this information is essential to support the breeding program of the Javan leopard, whose population is decreasing.

Therefore, this study aimed to develop electroejaculator (EE) stimulation techniques for semen collection and evaluate frozen Javan leopard semen quality with two different commercial extenders.

## Materials and Methods

### Ethical approval

The study was approved by the Animal Ethics Commission, Faculty of Veterinary Medicine, IPB University (number 185-2020 IPB).

### Study period and location

The study was conducted from April to May 2021. Four male Javan leopards (*P. pardus melas* Cuvier, 1809) from Taman Safari Indonesia (TSI) Cisarua, Bogor, West Jawa, Indonesia (location coordinates: S 6°42′53.1136″ and E 106°57′3.24746) were used in this study. The samples were processed at Taman Safari Indonesia, Cisarua, Bogor, Indonesia.

### Animals

The Javan leopards were estimated to be 8-12 years; three were captive-born and one was wild-caught. All animals were kept in individual 4×4×4 m cages and a paddock release area of 20×20 m. They were fed 8-10% of their body weight (BW) with chicken, kangaroo, or beef meat once a day with twice a week fasting. The Javan leopards were in good health with a body condition score of 3-4 with a 37-50 kg weight range; all leopards were weighed during every anesthesia before the EE procedure.

### Research methods

All EE procedures were conducted under anesthesia performed by TSI veterinarians. Animals fasted 12-24 h before anesthesia. Anesthesia drug protocol used a combination of ketamine hydrochloride (3 mg/kg BW) and medetomidine (0.05 mg/kg BW) administered intramuscularly through a service cage or blow syringe [21]. Animals were provided with blindfolds and nets during each semen collection process for safety. The animals were also given 0.1 mg/kg BW of meloxicam. An atipamezole reversal agent was administered 5 times with medetomidine dose subcutaneously at the end of the EE procedure. Anesthesia recovery is monitored in separate cages.

### Examination and measurement of the reproductive tract

Examination of the reproductive tract includes assessment and is performed in the left lateral recumbent position. Testicular consistency scores based on the elasticity level, testicular symmetry, and scrotal circumference measurements were performed using a measuring tape. Testicular consistency/elasticity scores were divided into five categories based on 1 (very firm), 2 (firm), 3 (moderate), 4 (soft), and 5 (very soft) [22].

The examination of internal reproductive organs was transrectally performed using ultrasonography (ultrasonography [USG]; ExaGo^®^, IMV imaging, USA). The USG probe used is a linear type 5-7.5 MHz for imaging and measuring prostate dimensions. Penile length measurements were conducted using a measuring tape and a penile spike score (penile spines). Visual observation was conducted to the penile spike, and the degree of smoothness of the penile surface for the presence of spines has a score of 1 (not visible or not palpable)-3 (very visible or detectable) [6].

### Semen collection using EE

Prepuce hair was cut and cleaned using saline solution then dried with a tissue. The fecal should be emptied from the rectum before EE probe insertion. A semen collection tube (15 mL, Corning^®^ Centrifuge tube, Corning, NY, USA) is attached to the exposed penis. The EE probe was lubricated with a USG gel and inserted through the rectum at 16-19 cm in a dorsoventral position pressing the prostate body, which had been seen on USG. The EE unit used is a 12 VDC EE (Ratek Instruments Mitcham^®^, Boronia, VIC, Australia).

The stimulation protocol was done by modifying the technique performed in the Sumatran tiger [23] and clouded leopard [11], which was conducted in as many as five stimuli at each voltage starting at 4.0, 4.5, 5.0, 5.5, and 6 V. Each trigger is given 5 s with a pause between impulses of 4.0-5.0 V of 5 s each, while 5.0-5.5 V with a pause of 10 s, and 5.5-6 V with a pause of 5 s. Erection and ejaculation reaction data were tabulated and descriptively presented.

### Semen evaluation

Semen evaluation was conducted using a semen examination procedure for carnivores [24]. The semen evaluation procedures consist of macroscopic and microscopic evaluation. The macroscopic evaluation was conducted on the semen volume, color, consistency, and degree of acidity (pH). Moreover, a microscopic evaluation was conducted on sperm motility, viability, concentration, and morphology [25]. The semen volume is measured directly on the scale indicated on the collection tube. The color of the semen can be visually seen. The consistency was observed with the watery, medium, and thick criteria. Furthermore, the acidity degree of the semen was determined using special pH indicator paper (6.4-8 scale).

This study conducted a sperm motility assessment by dropping 10 mL of semen and placing it on a warm slide glass and covered with a glass cover (without adding saline solution due to the low sperm cell count). Moreover, the slide was then viewed under a microscope (Olympus CX23) with 400×. At least five widely spaced fields are examined to provide an estimate of the percentage of motile cells. Sperm concentration was calculated using the Neubauer counting chamber. In addition, sperm viability examination was conducted by mixing 5 mL eosin nigrosin with 5 mL semen, mixed well, smeared, and dried on a heating table (37°C). Dead sperm has a high membrane permeability and will absorb the dead sperm. However, live sperm will not absorb the color.

### Semen cryopreservation

Javan leopard cryopreservation was performed on semen samples from three males with the highest sperm concentration. Two commercial extenders, AndroMed^®^ and Steridyl^®^ were used as cryopreservation media. AndroMed^®^ was prepared by mixing one part of AndroMed^®^ with four parts of Aqua Bidest (Otsu-WI^®^, Otsuka, Indonesia). Steridyl^®^ was prepared by mixing one part of Steridyl^®^ with three parts of Aqua Bidest. All extenders were then warmed at 37°C. Immediately after collection and evaluation, the ejaculate was divided into two parts (i.e., one part diluted with AndroMed^®^ and the second part with Steridyl^®^) at a 1:1 ratio. The semen was then packed in a 0.25 mL MiniStraw^®^ (Minitüb) and equilibrated at 4°C for 2 h [23]. The straw was then frozen in liquid nitrogen vapor after equilibration. Frozen semen is then stored in containers for further evaluation.

### Evaluation of frozen-thawed semen quality

The straw was thawed in warm water (37°C) for 30 s before evaluation. Thawed semen was then transferred into a microtube and stored at 37°C. The frozen semen quality test was performed using the computer-assisted semen analysis (CASA) platform Integrated Visual Optical System-II from Hamilton Thorne (Beverly, MA, USA) with HTCasa II software, ver. 1.12.1, and fitted with a video camera (JAI CM-040GE camera, JAI Ltd., Tokyo, Japan) with a frame rate of 60 frames per second and a final resolution of 776×582 pixels. The unit was equipped with an integrated microscope with Zeiss 10× NH 160 mm 10× negative phase contrast objective and an integrated heated stage maintained at 37±0.5°C. Semen quality examination using CASA was used to improve the evaluation of semen parameters [26]. Parameters taken using CASA include total sperm and progressive motilities. Sperm viability using eosin nigrosin staining was performed similarly to raw semen.

### Statistical analysis

The testicular circumference, testicular consistency score, penile spike, and penile length are presented in a table with averages. Prostate biometric data and semen quality, as well as post-clotting sperm motility and viability were processed with SPSS Statistics version 21.0 (IBM Corp., NY, USA) using a complete statistics randomized design. If a difference was noted, analysis followed using Duncan’s test. Data were presented in the form of mean and standard error of the means.

## Results

### Prostate biometry, testicular consistency, and scrotal circumference

**Figure-1:**
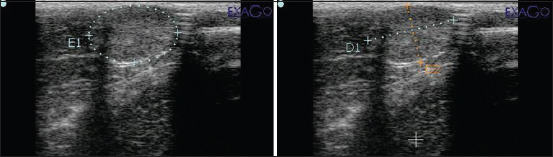
Imaging and measuring prostate circumference (E1), length (D1), and width (D2).

The ultrasound prostate imaging in this study showed oval-shaped uniform echogenicity with a smooth, mottled texture and a smooth hyperechoic capsular edge (Figure-1). The prostate gland of the felids is less developed than the canids and is smaller. The USG examination of the prostate size, testicular consistency, and scrotal circumference of the Javan leopard is shown in Table-1. The prostate length of leopards C and D was larger than that of leopard B (p<0.05). No difference in prostate length was noted between leopard A and leopards C and D and leopards A and B (p>0.05). The mean prostate length was 20.92±0.66 mm. The widest prostate was 16.2 mm found in leopard D, and the smallest was found in leopard B. The average prostate circumference was 224.17±44.40 mm, and the largest and shortest were found in leopards D and A, respectively.

**Table 1:** Biometry of the prostate, scrotal circumference, and testicular consistency score of the Javan leopard.

Parameter	Leopard				Mean±SE
	
	A	B	C	D	
Prostate gland					
Length(mm)	20.59±1.02^ab^	18.33±0.71^b^	22.33±1.16^a^	22.43±1.00^a^	20.92±0.66
Width(mm)	14.20±0.25^ab^	12.00±1.01^b^	13.90±1.54^b^	16.20±0.60^a^	14.08±0.61
Circumference(mm^2^)	232±20.82^ab^	174.33±23.88^b^	232.33±20.22^ab^	258±25.54^a^	224.17±13.39
Testicle and scrotum					
Testicle consistency scrotal	3±0	3±0	3±0	3±0	3±0
Scrotal circumference(cm)	14.5	14	13.5	14	14.00±0.41

Different letters following the numbers in the same row showed significant differences(p<0.05)

### Penile length and spines

**Figure-2:**
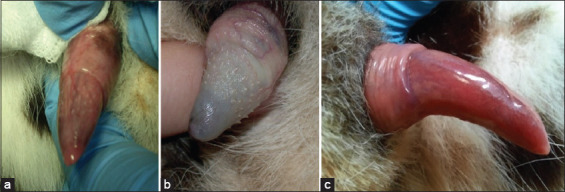
(a) Penile spike in the Arabian leopard [6], (b) other Panthera genera [6], (c) the penile spike in the Javan leopard.

The copulation organs are characterized by penile spines in Javan leopard and other Felidae families. The penile length data and spine scores are presented in Table-2. The average penile length of the four leopards was 3.03±0.40 cm with a penile spine score of 1. The penile of the four leopards was observed to be indistinguishable, but the spine pattern was smooth palpable in the penile gland (Figure-2) [6].

**Table 2:** Penile length and penile spike score Javan leopard.

Parameter	Leopard				Means±SE

	A	B	C	D	
Penile length	3.50	3.00	3.20	2.40	3.03±0.46
Penile spines score	1	1	1	1	1±0

### Response to EE stimulation in the Javan leopard

The response of EE stimulation to the erection and ejaculation parameters is different for each male. The erection process in all males is followed by ejaculation. Ejaculation in leopard A occurred at almost all stimulation levels. Erection was seen only at 4, 5, and 5.5 V. Erection in leopard B occurred at 4.5, 5, and 5.5 V. Moreover, erection in leopard C was seen at 4.5, 5.5, and 6 V. The incidence of erection in leopard D was found once at 4.5 V (Table-3). The ejaculation process in leopards occurred almost at all stimulation levels, except in leopards A and D, when no ejaculation was noted at the highest stimulation. The absence of ejaculation in leopards A and B may be due to semen emptying during the initial impulse. In the Javan leopard, an erection accompanies ejaculation; however, an erection does not follow ejaculation in some animals. The earliest ejaculation that happened without an erection in this study occurred in leopard A, appearing on the first second of stimulation. The reaction of the stimulus in this study started at 4 V.

**Table 3:** Electroejaculator response to erection and ejaculation.

Stimulation(Volt)	n	Leopard							

		A		B		C		D	
			
		Erect	Ejac	Erect	Ejac	Erect	Ejac	Erect	Ejac
4	1	─	√	─	√	─	√	─	√
	2	√	√	─	√	─	√	─	─
	3	─	√	─	√	─	√	─	─
4.5	1	─	√	√	√	√	√	√	√
	2	─	√	─	√	─	√	─	√
	3	─	√	─	√	─	√	─	√
5	1	√	√	─	√	─	√	─	√
	2	√	√	─	√	─	√	─	√
	3	─	√	√	√	─	√	─	√
5.5	1	√	√	─	√	─	√	─	√
	2	√	√	─	√	√	√	─	√
	3	─	─	√	√	─	√	─	─
6	1	─	√	─	√	─	√	─	√
	2	─	√	─	√	√	√	─	√
	3	─	─	─	√	─	√	─	─

The symbol(-) indicates that there is no reaction, the sign(√) indicates the appearance of a response. Erect=Erection, Ejac=Ejaculation

### Javan leopard semen characteristics

The semen characteristics of the Javan leopard are shown in Table-4. The mean semen volume was 0.79±0.11 mL. No difference was noted between individual males in semen volume. The semen color was cloudy white to clear. The semen consistency in all Javan leopard males is watery and is also related to the low sperm concentration in the semen. The degree of acidity (pH) of Javan leopard semen showed a value of 7.44±0.14. The average sperm motility and viability of the Javan leopard in this study were 67.08±0.96 and 74.62±1.33%. Furthermore, no difference in sperm motility and viability was noted between individual males (p>0.05). Sperm concentration was 66.02±20.41×10^6^ mL^−1^.

**Table 4:** Characteristics of Javan leopard semen collected using an electroejaculator.

Semen characteristics	Leopard				Means±SE

	A	B	C	D	
Semen volume(mL)	1.17±0.17	0.53±0.15	0.83±0.17	0.63±0.28	0.79±0.11
Color	Claudy white	Claudy white	Claudy yellowish	Claudy white	Claudy white
pH	7.37±0.17	7.30±0.38	7.63±0.23	7.47±0.15	7.44±0.11
Consistency	thin	thin	Thin	Thin	thin
Motile sperm(%)	66.67±1.67	68.33±1.67	66.67±1.67	66.67±3.33	67.08±0.96
Viable sperm(%)	74.19±3.16	76.23±2.54	76.32±0.66	71.74±3.90	74.62±1.33
Sperm concentration(×10^6^ mL−^1^)	72.67±28.99	32.75±8.03	28.67±18.89	130±67.69	66.02±20.41
Total concentration(×10^6^)	85.67±32.84	16.99±4.87	27.83±19.42	53.40±12.90	45.97±11.77
Normal sperm morphology	43.10±2.35ab	39.92±1.40ab	32.50±4.00b	47.3±5.36a	40.71±2.25

Different letters following the numbers in the same row showed significant differences-(p<0.05)

### Sperm motility and viability of frozen-thawed Javan leopard sperm in two different commercial extenders

Sperm total motility, progressive motility, and viability of frozen-thawed semen in AndroMed^®^ and Steridyl^®^ extenders did not show significant differences (Table-5). The average sperm total motility, progressive motility, and viability were 15.37%, 6.20%, and 47.05%, respectively. However, a significant difference in the sperm progressive motility was noted among individual males (Table-6). Sperm progressive motility was demonstrated by leopard D (8.00±0.85%) which was higher than leopard A (4.89±0.49%). However, no differences were noted between leopards A and B and leopards B and D. The cryopreservation of the Javan leopard semen has never been reported before.

**Table 5:** Total motility, progressive motility, and sperm viability of leopard frozen thawed in two different extenders.

Parameter	Extender		Average

	AndroMed^®^	Steridyl^®^	
Sperm total motility(%)	15.68±1.94	15.07±1.83	15.37
Sperm progressive motility(%)	7.07±0.74	5.34±0.68	6.20
Sperm viability(%)	45.83±2.50	48.28±2.09	47.05

**Table 6:** Frozen-thawed sperm motility of individual Javan leopard.

Leopard	Sperm motility-(%)	

	Total-(TM)	Progressive-(PM)
A	12.87±1.67	4.89±049b
B	16.20±2.87	6.34±1.12ab
D	16.20±2.87	8.00±0.85a

Different letters following the numbers in the same row showed significant differences(p<0.05)

## Discussion

The prostate is the most prominent accessory gland in the male reproductive tract [27]. Moreover, the prostate examination is generally conducted in the Canidae, whereas abnormalities are rarely found in Felidae because indications of this disorder are rarely reported in cats [28]. However, information regarding USG of the prostate in the Javan leopard is not available. Other researchers reported that the prostate of the clouded leopard (*N. diardi*) performed transrectally with the mean furthest and closest plane lengths being 12±1 and 10±1 mm, respectively [11]. The prostate biometry in leopards has not been previously reported. The Javan leopards in this study have an age range of 8-12 years old, which is a productive age for *Panthera* [2].

The testicular consistency scores of the four leopards were almost similar and included moderate with an evenly distributed elasticity level. Testicular consistency is an indicator of testicular function and activity as well as abnormalities. Domestic cat testes with very soft consistency are associated with both azoospermic and oligozoospermic conditions [29]. A strict testicular consistency indicates a pathological condition (e.g., fibrosis with inferior semen characteristics) [29]. Animals with a testicular score of 2-3 generally showed better semen quality than animals with a score of 4-5.

This spine condition is different from what was reported on a 19-year-old Arabian leopard [6]. The Arabian leopard has a fine texture of the penile spines pattern, so a possibility of success in the natural reproduction process was noted. The penile spine is vital, considering that female tiger, as in the feline family, generally has induced ovulatory type [29]. However, spontaneous ovulation can occur in felids, which is rarely reported [29]. The presence of the penile spine will induce ovulation during mating; therefore, fertilization can occur if the male deposited good semen during copulation.

The felids penile spine is correlated with sexual maturity status [30]. The penile spine develops due to continuous androgen hormone stimulation [31] or its regression which may occur under the influence of gonadotropin-releasing hormone immunocontraception [32] and in castrated males [29]. Ejaculation still occurs in males without an erection is due to the relationship between the erection reaction and separate ejaculation; that is, the ejaculation reaction can be preceded or not preceded by an erection [32]. Electroejaculator reactions in semen collections in domestic cats have been reported by Hapsari [24], where erections occurred at 1 V at 5 s of stimulation. Hapsari [24] used a domestic cat weighing only 3 kg, while the Javan leopard used in this study weighed between 37 and 50 kg. Another difference in domestic cats is the reactions that occur that are always consistently sequential, starting with the erection appearance and followed by ejaculation.

The ejaculation process occurs due to the electric stimulation on the hypogastric nerve, which causes the smooth muscle of the epididymis to contract. EE causes the release stimulation of sperm and fluid from the accessory glands to the urethra. Felids prostate and bulbourethral glands contain Na^+^ and Cl^−^ concentrations favoring sperm motility [33]. The Javan leopard’s ejaculation volume in this study was lower than other large wild cats. Snow leopard, the jaguar, and the Sumatran tiger had a semen volume of 2.9±0.2 [34], 6.25±0.86 [35], and 1.5±0.4 mL [23], respectively. The Indian leopard’s ejaculation volume was 1.57±1.26 mL, which was also higher than the semen volume of this study. The difference is related to the animal’s weight. The Indian leopard weighs 60.45±4.99 kg, while the Javan leopard is only 37-50 kg.

The relationship between semen volume and BW was also reported in the Arabian leopard, the smallest leopard subspecies [36], with a weight of 31 kg, resulting in a semen volume of only 0.55±1.26 mL [6]. The color of the semen comes from the semen plasma fluid secreted in the accessory glands. A transparent semen color indicates low sperm concentration. Javan leopard’s pH tends to be more alkaline compared with those reported in livestock (e.g., ram, buck, and bull semen) with a pH ranging from 6.4 to 7.4.

Variation values in sperm concentration were found to be very high within individuals or between collections of the same individual. This variation is also seen in the concentration of the total sperm per ejaculation. The total concentration value was obtained by multiplying the semen volume with sperm concentration. The total sperm concentration is between 16.99 and 85.66×10^6^ cells. Similar semen characteristics have been reported in other *P. pardus* spp. (e.g., the Indian [5] and Arabian leopards [6]).

The sperm abnormality of the Javan leopard was also very high. These studies found the highest percentage of normal sperm morphology in leopards D and C as the lowest (p<0.05). No difference was noted in the average sperm morphology values in leopards A, B, and C and with leopards A, B, and D. High sperm abnormalities were reported in the genera of *Neofelis* and *Panthera* [9,11,14]. The average sperm percentage in this study can be used as a baseline for semen characteristics of the Javan leopard. The high standard error means of the values for all semen quality parameters can be caused by the high individual variation in wild animals.

External stimuli (e.g., sight, smell, and physical contact) with estrus females will stimulate the hypothalamic-pituitary-testis axis (HPT axis) to initiate a series of physiological responses that target the testes and lead to increased production of androgens and testosterone [37]. Clouded leopards kept close to females had better semen quality than leopards held alone [38]. The theory is that good semen production is related to the presence and stimulation of females and the integration of the HPT axis regulated reproduction. This study kept all Javan leopards close to males and females in one building complex. Another factor that affects semen characteristics is nutrition. Nutrition plays a key role in animal health and reproduction, and improvement in nutrition management has shown a marked increase in semen production and quality [38]. The previous study that compared semen volume, concentration, and motility of sperm produced by EE collection and catheterization technique showed higher sperm motility values found in domestic cat semen collected by EE [39].

Cryopreservation of Indian leopard semen was reported in 2001 using TEST yolk buffer (TYB) with 8% (*v*/*v*) glycerol as an extender medium showing total motility of 32.14±9.14% [5]. Pukazhenthi *et al*. [9] reported the cryopreservation success in clouded leopard (*N. nebulosa*) sperm using TYB as extenders with post-thawed motility of 21-35.58%. Furthermore, Ha *et al*. [40] reported a study on semen cryopreservation of two leopard cats (*P. bengalensis*) using Tris egg yolk, showing that post-thawed motility is 19% and 24%. The TYB extenders usage for cryopreservation in two clouded leopards shows a low percentage of total and progressive motile, which was only 3% and 5% [11].

Various factors can affect the quality of frozen animal semen, including freezing protocols, extender formulas, and cryoprotectants substances [16]. Two extenders were used in this study (i.e., AndroMed^®^ contains soy lecithin, which has a similar function to egg yolk; Steridyl^®^ contains sterile egg yolk as a membrane coating material) and both extenders include Tris buffer, antibiotics, glycerol, minerals, and sugar. The results showed that AndroMed^®^ and Steridyl^®^ both showed the ability to protect the sperm plasma membrane well. These can be seen from the post-thawed sperm viability, which was not significantly different and relatively high. The low sperm motility is due to sugar content in the two commercial extenders that are not suitable or inappropriate for leopard sperm. Research about the chemical composition of leopard semen plasma is needed to determine the main sugar content in the semen plasma. Knowledge of the type of sugar in leopard semen plasma is fundamental; the current study can add sugar according to the native seminal plasma composition of leopard semen. The quality of frozen leopard semen in this study is low and unsuitable for artificial insemination; however, it can use assisted reproductive technologies such as intracytoplasmic sperm injection (ICSI).

## Conclusion

This study concludes the development of a semen collection technique using EE, which was preceded by imaging the prostate gland using ultrasound as a guide to the location of the EE probe, which is effective for semen collection in the Javan leopard. The response to EE stimulation varies between males. The Javan leopard semen characteristics showed low semen volume, moderate sperm motility and viability, low concentration, and morphology of normal sperm. Sperm progressive motility of frozen-thawed Javan leopard semen in AndroMed^®^ and Steridyl^®^ was low. However, the quality of frozen leopard semen in this study can be used for assisted reproductive technologies such as ICSI with sufficient sperm viability and low motility.

## Authors’ Contributions

BHM, RIA, and NWKK: Conceptualized and designed the experiment. BHM, RIA, AW, DRS, and VTY: Collected the samples, conducted laboratory examinations, collected data, and performed data analysis. JM: Supervised the research. TPN: Performed CASA data analysis. BHM, RIA, NWKK, and TPN: Conducted the literature review, wrote the first draft of the manuscript, and edited and revised the manuscript. All authors read and approved the final manuscript.
